# Mast cells and the liver aging process

**DOI:** 10.1186/1742-4933-10-9

**Published:** 2013-03-07

**Authors:** Fabio Grizzi, Giuseppe Di Caro, Luigi Laghi, Paul Hermonat, Paolo Mazzola, Diane D Nguyen, Saba Radhi, Jose A Figueroa, Everardo Cobos, Giorgio Annoni, Maurizio Chiriva-Internati

**Affiliations:** 1 Laboratory of Molecular Gastroenterology, Humanitas Clinical and Research Center, Rozzano, Milan, Italy; 2 Department of Internal medicine and Gene Therapy Program, University of Arkansas for Medical Sciences, Little Rock, AR, USA; 3 Department of Health Sciences, University of Milano-Bicocca, Milan, and Geriatric Clinic, San Gerardo Hospital, Monza, Italy; 4 Department of Internal Medicine, Division of Hematology/Oncology, Texas Tech University Health Sciences Center, Lubbock, TX, USA; 5 The Laura W. Bush Institute for Women's Health and Center for Women's Health and Gender-Based Medicine, Texas Tech University Health Sciences Center, Amarillo, TX, USA; 6 Division of Hematology and Oncology, Department of Internal Medicine, School of Medicine, Texas Tech University Health Sciences Center, Lubbock, TX, USA

**Keywords:** Mast cells, Liver, Ageing, Immunosenescence, Inflammation, Immune system

## Abstract

It has now ascertained that the clinical manifestations of liver disease in the elderly population reflect both the cumulative effects of longevity on the liver and the generalized senescence of the organism ability to adjust to metabolic, infectious, and immunologic insults. Although liver tests are not significantly affected by age, the presentation of liver diseases such as viral hepatitis may be subtler in the elderly population than that of younger patients.

Human immunosenescence is a situation in which the immune system, particularly T lymphocyte function, deteriorates with age, while innate immunity is negligibly affected and in some cases almost up-regulated.

We here briefly review the relationships between the liver aging process and mast cells, the key effectors in a more complex range of innate immune responses than originally though.

## Introduction

Nearly 13% of the inhabitants of the United States are aged more than 65 years; this percentage will increase substantially over the next 50 years [1].

Although it can refer to any time-related process, the term “aging” is commonly used for post-maturational processes that lead to diminished homeostasis and increased vulnerability. The altered homeostasis in older organisms is probably the result of a genetic program that determines responses to exogenous influences and increases the predisposition to illness and death.

There are five main characteristics associated with aging in mammals: *1*) increased mortality after maturation; *2*) changes in the biochemical and physical properties of tissues; *3*) a progressive decrease in physiological capacities; *4*) a reduced ability to respond adaptively to environmental stimuli; and *5*) increased susceptibility and vulnerability to disease [2-4].

It is now recognized that human aging is a complex phenotype resulting from the continuous, lifelong adaptation of the body to unrepaired molecular and cellular damage to the organism caused by a variety of external and internal agents [5].

**Figure 1:**
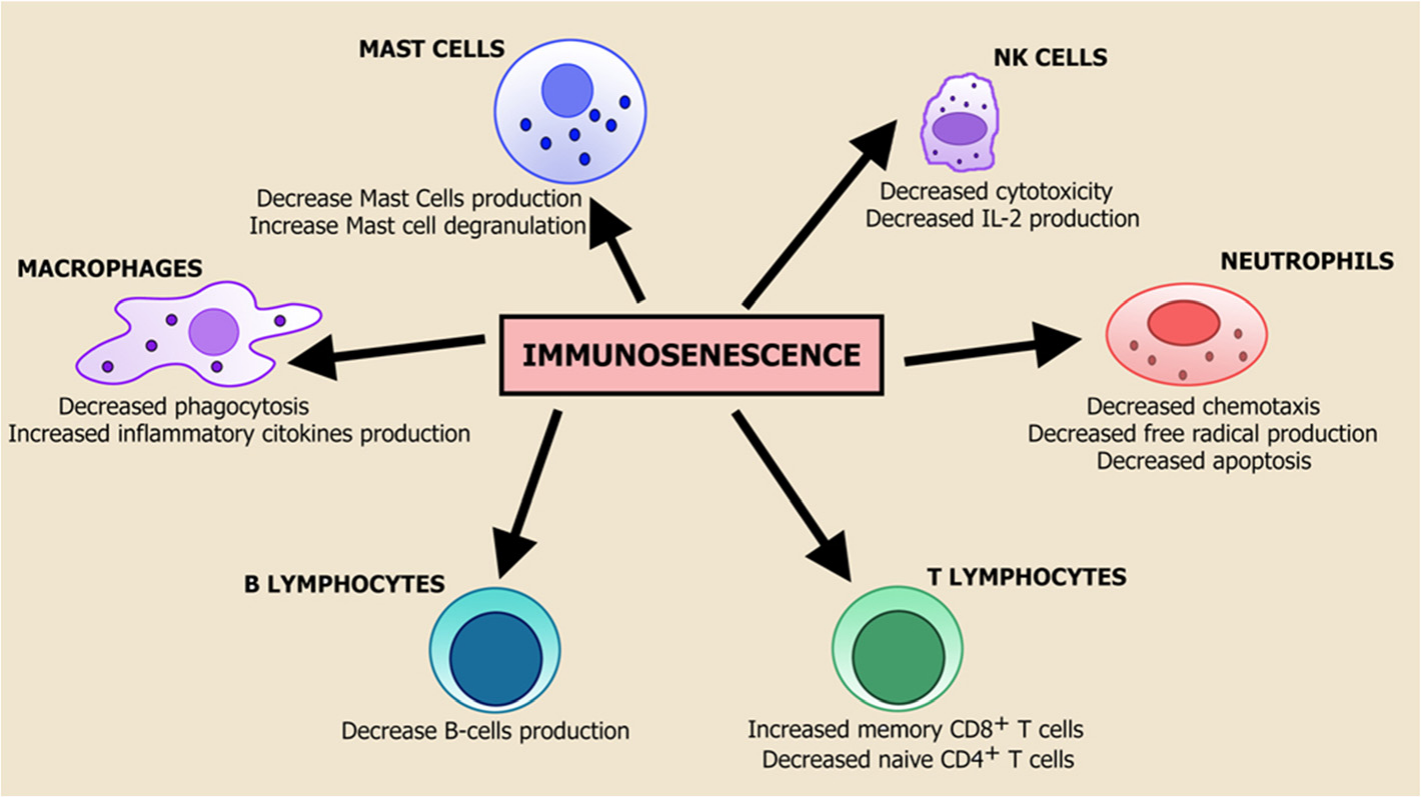
**Immunosenescence.** This phenomenon has been described as the result of a chronic hyperstimulation of the immune system components. Innate immunity is generally thought to be relatively well preserved or enhanced during aging compared with adaptive immunity, which is characterized by several alterations.

Several studies have highlighted that *immunosenescence* occurs as the result of a chronic hyperstimulation of both adaptive and innate immune system components [5-8], which together with the accumulation of molecular scars due to the progressive deterioration of molecular components and pathways [9] leads to a peculiar *immune status* characterized by a global loss of efficiency (Figure 1).

Although the human liver is not unscathed by the process of aging, the changes it undergoes are minor compared with other organ systems [10]. It has been ascertained that there are no liver diseases specific to advanced age. However, the clinical course and management of liver diseases in the elderly may differ in several aspects from those of younger adults [11]. We here briefly discuss the relationships between the liver aging process and mast cells (MCs), the key effectors in a complex range of immune responses.

### Aging and the immune system

Aging is associated with many physiological changes in a variety of organs [12]. It is today recognized that many diseases observed in the elderly have an immunological basis and are associated with the decline of immune response [13]. Clinically, the consequences of impaired immune function in the elderly include an increased susceptibility to infections, malignancies and autoimmunity [14-16]. It has been demonstrated that aging leads to the replacement of unprimed virgin T-lymphocytes by primed memory T-lymphocytes subpopulations and to the accumulation of cells with signal transduction defects [17]. T-lymphocytes are more severely affected than B-lymphocytes. This is mainly due to the involution of the thymus, which is almost complete at the age of 60 [18]. In humans, the thymus is a central lymphoid organ devoted to thymocyte differentiation and maturation, and is therefore the primary source of circulating T-lymphocytes. Although its size continues to increase until it reaches its maximum absolute weight during puberty, its functional compartments and T-lymphocytes output activity diminish after the first years of life onwards. Although it continues to serve as the site of T-lymphocytes differentiation and maturation throughout adulthood, the thymus undergoes a process known as *involution*, which is defined as a decrease in size, weight and activity of the gland with advancing age [19,20].

Other immunological cells, including macrophages, neutrophils, natural killer (NK) cells, and NKT lymphocytes, are affected by aging [21]. In addition, it has been shown that aged dendritic cells (DCs) are less able to stimulate T and B-lymphocytes, although the results of studies in humans and rodents focusing on DCs and aging are still conflicting.

Functional defects and altered frequencies of innate and adaptive immune cells impair local responses at the site of vaccine injection, hamper the generation of primary responses to neo-antigens, prevent the effective induction of memory lymphocytes, and decrease the effect of booster vaccination. As a result, antibody responses of elderly vaccines are weaker and decline faster, and long-term protective effects of vaccination cannot be taken for granted in elderly peoples [13,22-24]. Evidences exists that immunization procedures are less effective at older age than in young adults. Influenza virus vaccines have generally proved limited in preventing morbidity and mortality among the elderly because of the lower immunological protection that they may confer on older adults compared to younger persons [25]. Additionally, the measure of anti-hepatitis virus and anti-Hepatitis B surface antibodies (anti-HBs) in elderly people after a combined hepatitis virus A and B vaccination has underlined the decreased response to vaccination with increasing age [25].

It is now accepted that aging is characterized by a peculiar state of chronic inflammation (the so-called “inflamm-aging”) under genetic control, which seems to be a consequence of a person’s life-long antigenic load [26-30] and leads to a long-term tissue-related risk of all-cause mortality in older people [31,32]. A number of studies have highlighted that chronic inflammation is the underlying biological mechanism responsible for the decline in physical function observed in the elderly [33].

It has also been shown that aging is accompanied by a 2-4-fold increase in the plasma levels of inflammatory mediators, cytokines and acute-phase proteins [34-37]. Additionally, human aging is associated with a deregulated cytokine response following stimulation: chemotaxis and phagocytosis, as well as antigen processing and presentation, are all depressed in the elderly, whereas cell activation and the secretion of pro-inflammatory cytokines such as interleukin (IL)-1β, IL-6 and Tumor-Necrosis Factor (TNF)-α are markedly increased. Recently, it has been reported that inflammation in excess is detrimental, and that excessive production and secretion of cytokines may lead to pathology [25]. Kundu and Surh evidenced that a low-grade systemic inflammation characterizes aging and that inflammatory markers are significant predictors of mortality in old humans [38]. It is widely accepted that many aging-related diseases, including cardiovascular diseases, atherosclerosis, Alzheimer’s disease and diabetes, share a common inflammatory background.

### Aging and the liver

The incidence of liver disease increases with age while the ability to withstand a hepatic insult falls with each decade [39]. It is indubitable that interest in the role of aging in the sphere of hepatology has increased, especially with the recent recognition of the critical importance of age in determining the clinical outcome in chronic hepatitis C virus (HCV) infection and the influence of donor age on graft survival after liver transplantation [39]. Human and experimental studies suggest that, in comparison with other organs, the liver ages fairly well [40]. Aging is however associated with a variety of morphological changes in the liver [40,41], but their underlying mechanisms are still unclear. The liver progressively shrinks by 20-40% during the course of a human life [42-44], and there is a concomitant age-related decrease in liver volume [43,45]. The classic gross appearance of the liver in the elderly is known as “brown atrophy”, and the brown is due to an accumulation of highly oxidized insoluble proteins, known as lipofuscin, stored into hepatocytes. These accumulations of highly cross-linked protein are thought to relate to chronic oxidative stress and a failure to degrade damaged and denatured proteins [39]. Increasing evidence suggests that lipofuscin interferes with complex cellular pathways due to its ability to trap metallic cations and facilitate further free radical formation. Other sub-cellular hepatocyte changes with age involves a marked decline in smooth endoplasmic reticulum surface, which correlates with decreased hepatic microsomal protein concentrations and enzymatic activity including glucose-6-phosphatase; furthermore, the human liver tends to develop macro and polyploid hepatocytes with increased nuclei and nucleoli during aging [46]. Kudryavtsev et al. reported that after the age of 85, around 27% of human hepatocytes demonstrate polyploidy compared to around 6% for individuals in their twenties [47].

One of the most important age-related changes in liver function observed in animal models is a significant decrease in regenerative capacity of the liver, but not in the capacity to restore the organ to its original volume [48]. Liver regeneration in both young and old animals was complete by day 7 after 70% hepatectomy, but at day 1 younger animal had significantly increased liver mass and increased intrahepatic mitotic activity. It has also been shown that aging is associated with multiple changes in [49-53]. Elderly humans secrete less bile acid, have increased biliary cholesterol levels, and show an increased oxidative stress that is mainly attributable to a reduced capacity to eliminate metabolically generated superoxide radicals as efficiently as before [41]. The reduction in hepatic blood flow during aging reduces the metabolism of rapidly cleared drugs [54]. Aging of the liver is also associated with impaired metabolism of drugs, adverse drug interactions, and susceptibility to toxins [53,55]. Additionally, it is now accepted that drug [53] and pharmacodynamics may be altered in the elderly. An important contribution is made by decreased renal function, but biotransformation in the liver may also play a fundamental role [56,57]. Serum bilirubin and classical enzymes unchanged with aging [58]. However, sensitive tests of liver function have revealed a small decline with aging. This has been confirmed in experimental studies that have demonstrated changes in mRNA in the genes involved in cell stress and fibrosis [59]. Albumin synthesis decreased slightly with age, but this seems to be secondary to increased cytokine production owing to the levels of chronic inflammation [60].

Older people have a higher incidence of acute liver failure and a higher mortality with acute hepatitis A [61,62]. Along with the considerable growth in Western societies of relatively healthy elderly populations, it is inevitable that there will also be an increase in the number of elderly people with chronic liver diseases (mainly hepatitis C virus (HCV)-related cirrhosis, alcoholic cirrhosis and HCC).

Poynard et al. showed that age at HCV infection was a main risk factor for fibrosis [63,64]. The rate at which fibrosis progressed was low in individuals infected when younger than 20 years, intermediate in those infected at age 21 to 40 years, increased in those infected at age 40 to 50 years, and highest in those infected at 50 years of age or older. The reason for more rapid progression of liver fibrosis in older patients affected by HCV is still unknown, but seems to be related to the decline in immune function with age. Hartmann et al. showed that telomere shortening (the telomere length of somatic cells becomes shorter with aging because of the “end replication problem”) represents a causal factor impairing liver regeneration and accelerating cirrhosis formation in response to chronic liver disease [65].

Age is an independent risk factor for poor outcome in primary biliary cirrhosis (PBC) in addition to the presence of portal hypertension and impaired liver function [66-68]. It has been hypothesized that the increased susceptibility of aging people to neoplastic diseases are due to a decrease in basic immune defense functions [39,69].

It is well recognized that the incidence of HCC in patients with HCV correlates with progression of liver fibrosis. However, there is little information on the impact of aging on hepatocarcinogenesis. Furthermore, it is not known whether the putative etiologic factors and clinical and pathological features of HCC differ between young adults and older patients [70]. The elderly patients developed HCC more often, despite their lower grade of fibrosis, compared with the younger patients. Youssef et al. reported that the hepatocarcinogenic effect of Peroxisome Proliferator-Activated Receptor Alpha (PPAR alpha) agonists is enhanced by aging [71]. Exposure to these chemicals produces a five- to seven-fold higher yield of grossly visible hepatic tumors in old relative to young animals [71]. Newer evidence indicates an active age-related change in the regulation of malignant hepatocyte proliferation. Substantial attention has focused on the telomere/telomerase system as a mediator of replicative capacity. Telomeres are repeating hexanucleotide sequences that function to protect chromosomes against chromosomal end-end fusion and non-reciprocal translocations. Telomerase is a reverse transcriptase consisting of enzymatic (TERT), RNA template (TERC) and several other protein components including heat-shock protein 90 (hsp90) and dyskerin [39,72]. Telomerase can maintain telomere length by adding TTAGGG repeats, but its expression is controlled in differentiated and stem-cell populations [73-75]. Short human telomeres have been linked to the subsequent onset of disease and death in healthy older persons [76]. Kitada et al. [77] and Urabe et al. [78] showed that subjects with chronic viral hepatitis had shorter hepatic telomeres than healthy controls and that increasing fibrosis was associated with shorter telomere lengths. The role of the telomere/telomerase system in the pathogenesis of HCC have led some to suggest that therapeutic manipulation may hold the promise for future therapies [39]. Telomerase inhibitors are currently in phase II clinical trials and would represent a rational therapy for HCC. However, the potential for inducing fatal liver failure, given the results from mouse models, may limit their safety and efficacy.

### Mast cells: a heterogeneous innate immune cell population

Mast cells (MCs) have a rather unique position among cells of the immune response. Paul Ehrlich first described MCs in his 1878 doctoral thesis: he called them “mastzellen” (*maestung* - a root of the English word mastication; the active form “measten” is still in use) because of their characteristic staining of proteoglycan and protease-rich cytoplasmic granules. Ehrlich also noted the tendency of MCs to be associated with blood vessels, nerves, and glandular ducts.

It has been estimated that human MCs contain 2.4 to 7.8 μg heparin per 10^6^ cells [79]. This observation, along with the knowledge that heparin is a negatively charged molecule helps explain why MC granules are preferentially stained with cationic dyes [80]. In their traditional role, MCs are key players in immunoglobulin E (IgE)-associated immune responses via aggregation of the high-affinity IgE receptor, FcεRI, that is expressed on MCs as a heterotetrameric receptor with subunits that initiate specific signaling events [81]. Both positive and negative effects are elicited by FcεRI, which includes degranulation, gene transcription and eicasanoid production. More recently, it has been noted that MCs are not regulated solely by IgE-dependent mechanisms. New reports show that MCs express other surface receptor binding sites such as Toll-like receptors (TLRs), β2-integrins, intercellular adhesion molecule-1 (ICAM-1), androgen receptors, purinergic P2X receptors (P2X1, P2X4 and P2X7) and the serotonin receptor, 5-HT1A [81].

**Figure 2:**
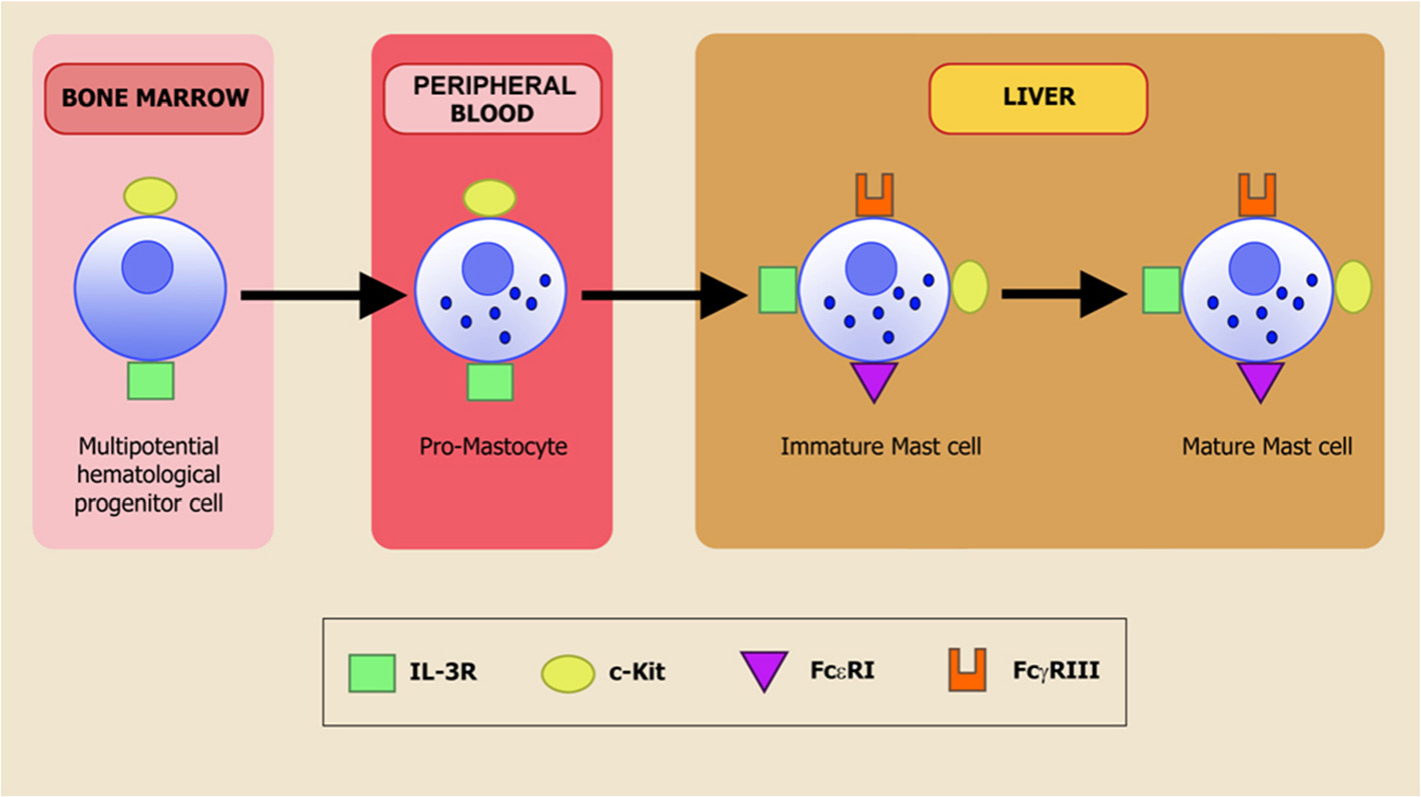
**Mast cells origin and differentiation.** It was initially suggested that Mast cells derive from T lymphocytes, fibroblasts or macrophages, but the current general consensus suggests that these immune cells originate from pluripotent hemopoietic stem cells in bone marrow, from where they are released into the blood as progenitors before they undergo terminal differentiation by invading connective or mucosal tissue as morphologically unidentifiable Mast cells precursors and then differentiating into phenotypically identifiable Mast cells.

It has long been recognized that MCs elicit allergic symptoms [82], but it is now widely accepted that they are multifunctional effector cells of the immune system, although the various phases of their differentiation are still only partially known. It was initially suggested that they derive from T lymphocytes, fibroblasts or macrophages, but the current general consensus suggests that they originate from pluripotent hemopoietic stem cells in bone marrow, from where they are released into the blood as progenitors before they undergo terminal differentiation by invading connective or mucosal tissue as morphologically unidentifiable MC precursors and then differentiating into phenotypically identifiable MCs [83] (Figure 2). It has been demonstrated that MC are long-lived cells [83].

MCs are found in almost all of the major organs of the human body, and in several body sites that come into contact with the external environment, including the skin, respiratory system and digestive tract. These main accumulations in sites where foreign material attempts host invasion suggest that MCs are one of the first cell populations to initiate defense mechanisms.

The ability of the lineage to generate individual MC populations with different biochemical and functional properties gives them greater diversity and flexibility in meeting the requirements of the physiological, immunological, inflammatory or other biological responses in which they may be involved [84-88].

A number of Authors have identified a local or systemic increase in the number of MCs in various pathological conditions, including interstitial pneumonia, ulcerative colitis, intestinal helminthosis and ectodermal parasitosis, as well as skin disorders such as atopic dermatitis, psoriasis, scleroderma and wound healing, and various neoplastic diseases [89-92].

Tryptases and chymases are the major proteases components of MC secretory granules. There are two well-known human MC classes that are distinguished by enzymatic immunostaining for mast cell proteases, tryptase (M_CT_) alone or tryptase and chymase together (MC_TC_). M_CT_ are found mainly in mucosal sites whereas MC_TC_[93]. These two populations of MCs exhibit functional differences [84]. TNF was the first cytokine clearly associated with normal MCs in 1990 [87,92]. Other MC products include interleukins (IL-3, IL-4, IL-5, IL-6, IL-9, IL-10, IL-11, IL-12, IL-13, IL-15, IL-16, IL-18, IL-2), chemokines (macrophage inflammatory protein alpha [MIP-1]), hematopoietic factors (granulocyte macrophage colony stimulating factor [GM-CSF]), stem cell factor (SCF), growth factors (transforming growth factor beta [TGF-β], vascular endothelial growth factor [VEGF], nerve growth factor [NGF]), several metalloproteinases, heparin, histamine, chondroitin sulfates, cathepsin, carboxypeptidases and peroxidase [84,87].

These products may be released when MCs are activated via IgE- or IgG-dependent mechanisms, and may also be produced under other circumstances such as in response to stimulation by bacterial products through Toll-like receptors (TLRs) [94].

MCs and IgE have long been associated with the pathogenesis of the acute manifestations of the immediate hypersensitivity reaction, the pathophysiologic hallmark of allergic rhinitis, allergic asthma, and anaphylaxis. The central role of MCs in these disorders is now widely accepted. Additionally, MCs are considered to be critical effectors in many human inflammatory diseases, and the core of an immediate hypersensitive reaction: they have been incriminated in different diseases including allergy, asthma, rheumatoid arthritis, arteriosclerosis, chronic graft-versus-host disease, fibrotic disease, ischemic heart disease and malignancy, and contribute to the progression of chronic diseases [95-99]. Any alteration in cell programs that determines a requirement for MC degranulation may therefore have a considerable impact on disease severity. Nguyen et al. have shown the ability of Prostaglandin E2 (PGE_2_) to initiate MCs degranulation changes in the aging animal, thus suggesting that aging induces reprogramming of MC degranulation [100].

**Figure 3:**
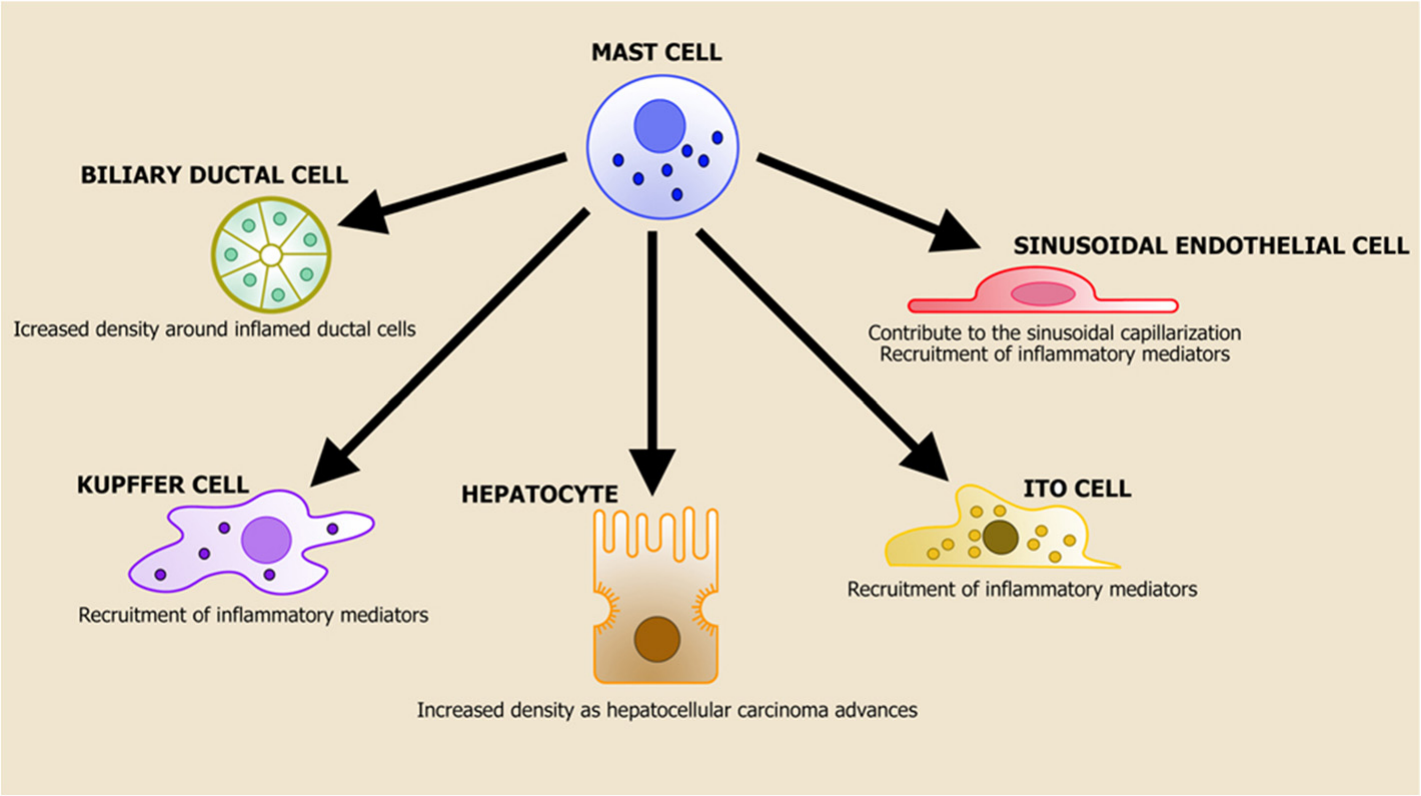
**Mast cells and the liver cell interactions.** The parenchyma of the liver is a complex structure with different cell types having various functions in normal liver function and disease response. These include: macrophages, or Kupffer cells, hepatic stellate cells, sinusoidal endothelial cells, vascular endothelial cells, fibroblasts and pit cells. It has been ascertained that all these cells (in addition to biliary cells) play definitive roles in liver pathophysiology and determine complex interactions with hepatic MCs.

The parenchyma of the liver is a complicated structure with numerous cell types having different functions in normal liver function and disease response. These include: Kupffer cells, hepatic stellate cells, sinusoidal endothelial cells, vascular endothelial cells, fibroblasts and pit cells. The majorities of these cells play definitive roles in liver pathophysiology and interact with hepatic MCs (Figure 3). MC quantity has been shown to increase in PBC by staining for either toluidine blue or immunohistochemical staining for tryptase and chymase, evidence that these cells are presumably MC_TC_. Increased plasma histamine levels were also found in patients with PBC, in contrast to healthy patients, raising the possibility of a role for in vivo mast cell activation and mediator release in these diseases [101]. Hepatic MC expression and quantity have been also shown to increase in patients with primary sclerosing cholangitis (PSC) compared to healthy controls. Increased MC infiltration has been noted in bile ducts in patients with early stage PSC with a diffuse staining in the fibrous septa in the late stage of PSC [102].

### Mast cells and liver aging

It has been recognized that liver MCs are present under normal and pathological conditions in both humans and experimental animals [87]: normal human livers have only ≈1.2-3.9 MCs/mm^2^, but rat livers have 1.8-12 MCs/mm^2^[87]. Immunostaining with antibodies against chymase or tryptase, or metachromatic dyes, has shown that hepatic MCs are mainly associated with the connective tissues adjacent to hepatic arteries, veins and bile ducts of the portal tracts [87].

MCs seem to be involved in the liver’s fibrotic response to chronic inflammation [103] and parasitic infection as different studies have established that they can induce liver fibrosis in humans and animal models following liver damage caused by autoimmune reactions, chemical toxins, viral infections and cholestasis [87]. However, it has also shown that MCs play no primary role in liver fibrosis in mast cell-deficient rodents [87] and, as there is no significant relationship between chymase activity in liver tissue and the severity of liver fibrosis, the role of MCs in liver fibrogenesis remains a subject of controversy [87].

MCs infiltrating portal tracts and surrounding damaged bile ducts have also been found in patients with chronic allograft rejection, thus suggesting that they may be important effector cells in the pathogenesis of chronic transplanted liver rejection [87]. The liver is also affected during systemic mastocytosis, a clonal disorder of MCs and their progenitors that is characterized by abnormal MC proliferation in different organs [80,87]. Finally, it has been associated MCs and angiogenesis in liver neoplasia because the significant correlation between MCs and microvessel density suggests they may play a role in tumor progression by promoting angiogenesis [90].

MCs have recently been investigated in order to study their involvement in the mechanisms leading to age-related chronic diseases. During fibrosis and hepatic disease progression (i.e. hepatitis), hepatocytes maintain a close interaction with other resident cell types as well as inflammatory cells. These populations come together to induce “sinusoidal capillarisation” which describes the events that cause the sinusoids to resemble capillaries. Capillarisation inhibits the normal processes of exchange between hepatocytes and plasma and has been found to be a condition that worsens liver function. It has been showed that liver sinusoidal endothelial cells become thicker and defenestrated, there is a sporadic deposition of collagen and basal lamina in the extra-cellular space of Disse, and the number of non-activated stellate or Ito cells increases [41]. This pseudo-capillarization leads to relative hypoxia of hepatocytes in older rats and may be reflected by lower ATP/ADP ratios in older versus younger rats [41,54,55,57]. In humans, these morphological changes have potential clinical implications, including adverse drug reactions and acute hepatitis [104-109].

In old age, there is a 30-50% reduction in the area of the endothelium perforated by fenestrations (“porosity”). This is associated with increased endothelial thickness and extracellular matrix in the space of Disse, including collagen and basal lamina [41,110-112]. These ultrastructural changes are associated with increased expression of antigens not usually expressed in young healthy livers such as von Willebrands factor and collagen IV. DeLeve et al. [113] demonstrated that sinusoidal endothelial cells prevent hepatic endothelial cells activation and also promote reversion of activated hepatic endothelial cells to a quiescent phenotype, whereas capillarized sinusoidal endothelial cells lose this effect. Coupled with the in vivo observation in humans and in animal models that capillarization precedes fibrosis, the findings in this study suggest that capillarization of sinusoidal endothelial cells may be permissive for hepatic endothelial cell activation and fibrosis. A role for mast cells in this process has been investigated. Studies suggest that mast cells contribute to capillarisation by recruiting other liver matrix-producing cells, thus increasing the secretion of cytokines and other mediators during the progression of liver fibrosis [87]. A variant of capillarization called pseudocapillarization is seen with aging [110-112].

It has been shown that MC density increases with age [114-120] but, although human and experimental studies have revealed many of the roles played by MCs in physiological and pathological conditions, there is still debate concerning their function as the critical initiators of inflammatory reactions, and whether they can be considered an early index of acute liver injury.

Recently it has been evaluated MC density in liver tissues taken from untreated and CCl_4_-treated young (2 months), middle-aged (6 and 12 months) and old male rats (19 months) as a quantitative index of acute toxic liver inflammation, and investigated whether the density is age-dependent [121,122].

Histological examinations of the 12- and 19-month-old rats sacrificed two hours after CCl_4_ intoxication showed hepatocyte necrosis and inflammatory cell infiltration in the perivenular areas, whereas the periportal areas were virtually unaffected.

It was found no statistical differences in MC density between the untreated rats of different ages, but MC density was considerably increased in the young rats both two and 24 hours after CCl_4_ intoxication, with the 24-hour difference being statistically significant. The increase in MC density was less marked in the 19-month-old rats than in the young rats, but there were significant differences between the untreated rats and the treated rats two hours after CCl_4_ intoxication, and between the densities measured two and 24 hours after intoxication. The changes in the rats aged six and 12 months fell between those observed in the rats aged two and 19 months.

It is well known that MCs are a primary cell population involved in inflammatory responses. By means of the rapid release of their pro-inflammatory molecules, MCs determine a series of events that lead to both immediate and late-phase responses.

Quantitative analysis of MC density in untreated rats and rats receiving an intraperitoneal injection of CCl_4_ highlighted the fact that MC density is an important marker of an acute liver inflammatory reaction to toxic CCl_4_ damage: it considerably increased two hours after intoxication (which suggests that MC cells are recruited early in injured tissue) and the increase was even more evident after 24 hours, when the density was significantly greater than in the control animals [121]. As MC density two and 24 hours after intoxication was greater in the young than in the oldest rats, this suggests that the latter have fewer MC recruiting stimuli and that young rats are characterized by a rapid response to toxic injury. This different behavior is in line with theoretical models showing that a large number of biological events are age-dependent. Future studies of inflammatory reactions could investigate the relationships between MC biology, age and functional activities (metabolism) in human patients. In fact, although most theories of aging assume that cell functions decline with aging, many intracellular functions in the liver, such as enzyme activities, stay fairly stable in old age.

## Conclusions

It is indubitable that aging, being a complex process, involves an array of intertwined molecular pathways [123,124]. Simultaneous study of multiple molecular pathways in parallel could provide invaluable information in understanding the clinical course of liver aging and elucidating mechanisms that play key roles in the aging process. Recent observations indicate that immuno-senescence is the result of a remodeling where some functions are reduced, others remain unchanged or even increased. In addition, it appears that the non-adaptive compartment of the immune system is relatively preserved during aging in comparison to the more recent and sophisticated adaptive compartment that exhibit more profound modifications [125]. On the basis of the above observations, the following conclusions are made: a) the incidence of liver disease increases in the elderly, but the cellular and sub-cellular perturbations underlying this predisposition to pathology remain still unresolved. b) Although the liver ages fairly well, several age-related morpho-functional changes have been highlighted, including: *a*) a decline in liver volume; *b*) an increase in the hepatic pigment (lipofuscin) deposition; *c*) a moderate decline in the Phase I metabolism of certain drugs; *d*) shifts in the expression of a variety of proteins and *e*) diminished hepatobiliary functions. Other more subtle changes may contribute to increased susceptibility to certain liver diseases in the elderly [126]. *e*) Aging of the liver mainly affects the sinusoids. Pseudo-capillarization, manifested by reduced sinusoidal fenestration and subendothelial collagen deposition, causes a reduction in oxygen-dependent hepatocyte functions such as oxidative drug metabolism [127]. *f*) Reduced hepatic blood flow in the elderly has been suggested to be the major effect of aging on the liver circulation. *g*) MCs can exert positive or negative immunomodulatory effects on immune cells by influencing the recruitment, survival, development, phenotype or function of immune cells and thereby enhance or suppress the initiation, magnitude and/or duration of immune responses [128]. *h*) Quantitative analysis of MC density in liver specimens taken from untreated rats and rats receiving an intraperitoneal injection of CCl_4_ highlighted the fact that MC density is an important marker of acute liver inflammation. *i*) MC density two and 24 hours after CCl_4_ intoxication was greater in the young than in the oldest rats, which suggests that the latter have fewer MC recruiting stimuli and that young rats are characterized by a rapid response to toxic injury.

In conclusion, a better understanding of the mechanisms underlying the age-related liver changes may help to preserve hepatic function, improve morbidity and mortality, and hopefully reduce healthcare costs for the aging population. Additionally, it is likely that future studies of MCs in experimental models and human tissues will reveal more about their functions not only in the pathogenesis of liver and other diseases, but also in the immunosenescence process affecting human tissues.

## Abbreviations

CCl4: Carbon tetrachloride; DCs: Dendritic cells; FcεRI: High-affinity IgE receptor; GM-CSF: Granulocyte macrophage colony stimulating factor; HCC: Hepatocellular carcinoma; HCV: Hepatitis C virus; hsp90: Heat-shock protein 90; ICAM-1: Intercellular adhesion molecule-1; IL: Interleukin; MCs: Mast cells; MCT: Tryptase; MCTC: Tryptase-chymase; MIP-1: Macrophage inflammatory protein alpha; NGF: Nerve growth factor; NK: Natural killer cells; NKT: Natural killer T-lymphocytes; PBC: Primary biliary cirrhosis; PGE2: Prostaglandin E2; PPAR: Peroxisome proliferator-activated receptor; PSC: Primary sclerosing cholangitis; SCF: Stem cell factor; TERC: Telomerase RNA component; TERT: Telomerase reverse transcriptase; TGF: Transforming growth factor; TLR: Toll-like receptor; TNF: Tumor necrosis factor; VEGF: Vascular endothelial growth factor.

## Competing interests

The authors declare that they have no competing interests.

## Authors’ contribution

FG and MCI wrote the first draft; subsequent drafts were written by FG, GDC, LL, EC and MCI who had the overall supervision of the review processing. All authors read and approved the final manuscript.
